# 

**Published:** 1965-07

**Authors:** 


BRISTOL MEDICO-CHIRURGICAL JOURNAL

*(Incorporating the Medical Journal of the South-West)*

Established 1883

VOL. 80 (iii) July 1965 NO. 297

### Contents

KENNETH PRIDIE: AN APPRECIATION *A. L. Eyre-Brook* 37

FAMILIAR NEUROSURGICAL PROBLEMS *Douglas G. Phillips* 42

PLACENTAL LOCALIZATION BY ARTERIOGRAPHY
*M. B. Wingate and N. D. O'Connell* 50

CYTOMEGALIC INCLUSION-BODY DISEASE
*Clinico-Pathological Conference* 53

APPOINTMENTS . . . . . . . . . . 57

*PUBLISHED QUARTERLY*

*ANNUAL SUBSCRIPTION* **16/-** *POST FREE*

BRISTOL MEDICO-CHURURGICAL JOURNAL

PUBLISHED UNDER THE AUSPICES OF THE

### *Bristol Medico-Chirurgical Society*

The Society was founded in 1874. In 1883 publication of this Journal began, and has continued quarterly since then. During the years 1949 to 1962 it appeared under the title of "The Medical Journal of the South West".

The Society founded a medical library in 1890, which in 1892 was combined with that of the Bristol Medical School. This became the University of Bristol Medical Library in 1925, and an agreement in that year confirmed the privilege of Members to use the University Medical Library.

### *Editorial Committee*

Dr. N. J. Brown, Southmead Hospital, Bristol. *Joint Editor.*

Dr. A. B. Raper, Department of Pathology, Bristol Royal Infirmary. *Joint Editor.*

Dr. J. Macrae, Ham Green Hospital.

Dr. R. C. Wofinden, Central Clinic, Bristol.

Mr. A. E. S. Roberts, Medical Library, University of Bristol. *Secretary.*

### *Local Correspondents*

*Bath* . . Mr. A. D. Bateman, 3 The Circus, Bath.

*Exeter* . . Dr. L. N. Jackson, Mount Jocelyn, Crediton, Devon.

*Gloucester* . Dr. R. F. Jarrett, 9 College Green, Gloucester.

*Plymouth* . Dr. C. R. Croft, Lexden, Hartley Av., Plymouth.

*Taunton* . Dr. M. McAlley, 1 The Crescent, Taunton.

*Torquay* . Dr. A. Robinson Thomas, 20 Devon Square, Newton Abbot, Devon.

*Truro* . . Dr. F. D. M. Hocking, St. Andrews, Perranporth, Cornwall.

*Yeovil* . . Mr. J. A. Vere Nicoll, Knapp House, Yeovil, Somerset.

### NOTICE TO CONTRIBUTORS

**Original articles are invited, provided they have not been published elsewhere. Notes of forthcoming events, meetings held, degrees conferred, staff appointments, and other local news are welcomed. The editorial committee reserves the right to make literary corrections.**

**Illustrations should always be supplied ready for reproduction. All re-touchings or other operations required will be charged to the author. Line drawings should be drawn in Indian ink. We regret that we must ask authors to pay for making blocks, if this is necessary.**

J. W. ARROWSMITH LTD., WINTERSTOKE ROAD

BRISTOL.

### *Appointments*

### SOUTH WESTERN REGIONAL HOSPITAL BOARD
### CONSULTANTS, SPECIALISTS AND REGISTRARS APPOINTED DURING 1964

| *Name* | *Appointment* |
|---|---|
| Craig, J. K., M.B., CH.B., F.R.C.S., M.R.C.O.G. | Consultant Obstetrician and Gynaecologist, Bath |
| Yeoman, P. M., M.A., M.D., B.CHIR., F.R.C.S. | Consultant Orthopaedic Surgeon, Bath. |
| Nairac, M. L., M.R.C.S., L.R.C.P., D.T.M. & H., D.O.M.S. | Consultant Ophthalmologist, North Gloucestershire. |
| Shaw, D. B., M.D., B.S., M.R.C.P. | Consultant Physician, Devon and Exeter. |
| Paine, M. G., M.B., B.S., D.M.R.T. | Consultant Geriatrician, North Gloucestershire. |
| Griffiths, H. E. D., M.B., B.S., F.R.C.S. | Consultant Orthopaedic Surgeon, Bristol. |
| Wright, W. B., M.B., CH.B., M.R.C.P. (Edin.) | Consultant Geriatrician, Devon and Exeter. |
| McAneny, T. M., B.SC., M.B., B.S., D.A., F.F.A.R.C.S | Consultant Anaesthetist, Bristol. |
| Whittard, B. R., M.B., B.S., F.F.A.R.C.S. | Consultant Anaesthetist, Torquay. |
| Wakefield, G., M.B., B.S., M.R.C.P. | Consultant Neurologist, Bath. |
| Davidson, G. M., M.B., CH.B., M.R.C.O.G. | Consultant Obstetrician and Gynaecologist, Plymouth. |
| Murrell, J. S., M.B., B.S., M.R.C.P. (Edin.) | Consultant Pathologist, West Cornwall. |
| Nicol, A., M.D., CH.B. | Consultant Pathologist, North Gloucestershire. |
| Wardle, C. J., M.D., B.S., D.P.M. | Consultant in Child Psychiatry, Devon and Exeter. |
| Fraser, I. D., M.D., CH.B. | Consultant Pathologist, Cheltenham. |
| Gossmann, H. H., M.B., B.S., F.R.C.S. (Edin.) | Consultant Neurosurgeon, Plymouth. |
| Turner, M. J., M.B., B.CHIR., D.M.R.D., F.F.R., M.R.C.P. (Edin.). | Consultant Radiologist, Torquay. |
| Nesling, A. E., M.B., CH.B., F.F.A.R.C.S. (Eng.). | Consultant Anaesthetist, Plymouth. |
| Mayall, G. F., M.A., M.B., B.CHIR., D.M.R.D., F.F.R. | Consultant Radiologist, Devon and Exeter. |
| Francis, J. G., M.B., B.S., D.OBST.R.C.O.G., D.A., F.F.A.R.C.S. | Consultant Anaesthetist, Devon and Exeter. |
| Smith, E. B. O., M.B., B.S., M.R.C.P. (Edin.), D.P.M. | Consultant Psychiatrist, Roundway Hospital. |
| Covell, K. J., M.B., B.S., D.T.M. & H,D.A., F.F.A.R.C.S. | Consultant Anaesthetist, South Somerset. |
| Kirkup, J. R., B.A.M.B., B.CHIR., F.R.C.S. | Consultant Orthopaedic Surgeon, Bath. |
| Waters, D. J., B.M., B.CH., D.A., F.F.A.R.C.S. | Consultant Anaesthetist, North Gloucestershire. |
| Langley, G. E., M.B., B.S., M.R.C.P. (Edin.), D.P.M. | Consultant Psychiatrist, Devon and Exeter. |
| Williams, E. H., M.A., M.B., B.CHIR., M.R.C.P., F.F.R., D.M.R.D. | Consultant Radiologist, Weston-super-Mare. |
| Adam, R. M., M.B., B.S., M.R.C.O.G. | Consultant Obstetrician and Gynaecologist, South Somerset. |
| Mills, P. V., B.SC., M.B., B.S., F.R.C.S., D.O. | Consultant Ophthalmologist, Bath. |
| Hall, Elizabeth W., M.B., B.S. | Consultant Pathologist, Bath. |
| Lloyd Williams, K., M.A., M.D., M.CHIR., F.R.C.S. | Consultant Surgeon, Bath. |
| John, A. H., M.B., B.S., L.R.C.P., M.R.C.S., F.R.C.S.E., M.R.C.O.G. | Consultant Obstetrician and Gynaecologist, United Bristol Hospitals/Regional Hospital Board, Bristol. |
| Thould, A. K., M.D., B.S., M.R.C.P. | Consultant Physician, West Cornwall. |
| Waldron, B. le G., B.A., M.B., B.CHIR., D.A., F.F.A.R.C.S. | Consultant Anaesthetist, Plymouth. |
| Taylor, S. H., B.SC., M.B., CH.B., M.R.C.P. (Edin.) | Consultant Physician, Torquay. |

### *BRISTOL*

| | |
|---|---|
| Mitchell, A. R. K., M.B., CH.B., M.R.C.P. (Edin.). | Senior Registrar in Psychiatry, Glenside and Barrow Hospital Group |
| Douglas, H. G. K., M.B., CH.B., D.OBST., R.C.O.G. | Registrar in Obst. and Gynae., Southmead Hospital. |
| Hyland, J., B.A., M.B., B.CH., B.A.O., D.A. | Registrar in Anaesthetics, Southmead/Frenchay Hospitals. |
| Morphew, J. A., M.B., CH.B. | Registrar in Psychiatry, Glenside and Barrow Hospital Group. |
| Ajabor, L. N., M.B., CH.B., D.OBST., R.C.O.G. | Registrar in Obst. and Gynae., Southmead/Frenchay Hospitals. |
| Katiyar, B. C., M.D. (Lucknow) M.R.C.P.E. | Medical Registrar, Frenchay Hospital. |
| Thompson, C. E. R., M.A., M.B.B.CHIR., F.R.C.S. (Ed.). | Surgical Registrar, Frenchay Hospital. |
| Ogden, A. H., M.B.CH.B. | Registrar in Psychiatry, Glenside and Barrow Hospital Group. |
| Wallen, G. D. P., M.B.CH.B., D.OBST.R.C.O.G. | Registrar in Psychiatry, Glenside and Barrow Hospital Group. |
| Jones, G., M.B.B.CH., F.R.C.S., D.M.R.D. | Senior Registrar in Radiology, Southmead Hospital/United Bristol Hospitals |
| Black, J. H. A., M.B.B.CH., B.A.O., D.R.C.O.G. | Registrar in E.N.T. Surgery, Southmead/Frenchay Hospitals. |
| Verghese, A., M.B.B.S. (Madras), F.R.C.S. | Surgical Registrar, Weston-super-Mare General Hospital. |

### *NORTH GLOUCESTERSHIRE*

| | |
|---|---|
| Hall, R., B.SC., M.B.B.S. | Surgical Registrar, Cheltenham General Hospital. |
| Mucklow, E. S., M.A., B.M., B.CH., D.OBST., R.C.O.G., D.C.H. | Medical Registrar, Cheltenham General Hospital. |
| Mayne, Madaline, M.B., CH.B. | Medical Registrar, Gloucestershire Royal Hospital. |
| Ranney, D. A., B.A., M.D., (Toronto) | Surgical Registrar, Gloucestershire Royal Hospital. |
| Brayshaw, J. A., M.R.C.S., L.R.C.P. | Clinical Assistant in General Surgery, (two weekly sessions) Stroud General Hospital. |
| Mould, J., M.B.CH.B. | Clinical Assistant in General Surgery, (two weekly sessions) Stroud General Hospital. |
| Shillito, P. W., M.R.C.S., L.R.C.P. | Clinical Assistant in Orthopaedic and Traumatic Surgery, (two sessions) Stroud General Hospital. |
| Lamb, R. W., M.B.B.S., D.OBST., R.C.O.G., D.A. | G.P. Anaesthetist, (two sessions) Stroud General Hospital. |
| Chohan, B. A., M.B.B.S. (Karachi) | Clinical Assistant in Ophthalmology, Cheltenham General Hospital (six sessions). |

### *BATH*

| | |
|---|---|
| Drysdale, I. S., M.B.B.S. | Registrar in Anaesthetics, Bath Group of Hospitals. |
| Nixon, J. M., M.B.B.CH., B.A.O. (N.U.I.) | (Re-appointed) Registrar in Obst. & Gynae. Bath Group of Hospitals. |
| Parker, C., M.B.CH.B. | Surgical Registrar, Bath Group of Hospitals. |
| Tidman, M. K., M.B.B.S., F.R.C.S. | Surgical Registrar, Bath Group of Hospitals. |
| Bourke, M., M.B.CH.B., D.C.H. | Clinical Assistant in Paediatrics, Royal United Hospital, Bath, (one session) (Re-appointed). |
| Bazeley, R. W., M.B.B.S. (Re-appointed)<br>Beviss, J. E., M.B.CH.B.<br>Burrowes, W. L., M.D., CH.B., M.R.C.P. (Ireland) (Re-appointed).<br>Ellison, D. J., M.B.CH.B., M.R.C.P. (Ed.) (Re-appointed). | Clinical Assistants in General Medicine, (four posts each for one weekly session) Bath Group of Hospitals. |

### SOUTH SOMERSET

| | |
|---|---|
| Kelly, N. J., M.B.B.CH., B.A.O. (Dublin), D.P.M. (Durham). | Senior Registrar in Psychiatry, Tone Vale Hospital. |

### DEVON AND EXETER

| | |
|---|---|
| Butterfield, A. R., M.A., M.B.B.CHIR., F.R.C.S. | Clinical Assistant in General Surgery (three sessions), Teignmouth Hospital. (Re-appointed). |
| Foster, G. S., M.B.B.S., M.R.C.O.G. (Re-appointed). | Clinical Assistant in Obst. & Gynae., Tiverton and District Hospital. (Two sessions). |
| Keen, J. H., M.B.B.S., D.C.H., D.OBST., R.C.O.G. | Medical Registrar, Royal Devon and Exeter Hospital. |
| Trapnell, J. E., M.A., M.B., B.CHIR., F.R.C.S. | Senior Surgical Registrar, Royal Devon and Exeter Hospital/Southmead Hospital/United Bristol Hospitals. |
| Dawson, P., L.MED.L.CH. (Dublin). | Anaesthetic Registrar, Torbay Hospital. |
| Hamilton, S. G. I., M.A., M.B.B.CHIR., F.R.C.S. | Surgical Registrar, Royal Devon and Exeter Hospital. |
| Pakrashi, B. C., M.B.B.S. (Calcutta), M.R.C.P. (Ed. and Glasgow). | Medical Registrar, Torbay Hospital. |

### PLYMOUTH

| | |
|---|---|
| Roy, C., M.B.B.S. (Calcutta), D.P.M. (Durham). | Senior Registrar in Psychiatry, Moorhaven Hospital. |
| Clarence, Gilliam A., M.B., B.S., L.M.C.C. (Canada), D.OBST., R.C.O.G., D.C.H. | Paediatric Registrar, Plymouth General Hospital/ United Bristol Hospitals. |
| Hazelwood, J. G., M.B.CH.B. | Orthopaedic Registrar, Plymouth General Hospital/Mount Gould Orth. Hospital. |

### WEST CORNWALL

| | |
|---|---|
| Qureshi, L. A., M.B.B.S. (Punjab), D.C.H. | Medical Registrar, Redruth and Tehidy Hospitals. |
| El-Jamal, M. Z., M.B.CH.B. | Registrar in Obst. & Gynae., Camborne-Redruth Hospital. |

### UNITED BRISTOL HOSPITALS

| | |
|---|---|
| Keen, G., M.B., M.S., F.R.C.S. | Consultant Thoracic and Cardiac Surgeon. |
| Ratliff, A. H. C., M.B., CH.B., F.R.C.S. | Consultant Orthopaedic Surgeon. |
| Marmion, V. J., M.B., B.CH., B.A.O., F.R.C.S.E. | Consultant Ophthalmic Surgeon. |
| Burton, G. W., B.SC., M.B., CH.B., F.F.A.R.C.S., D.A. | Consultant Anaesthetist. |
| Holderness, M. C., M.B., CH.B., F.F.A.R.C.S., D.A. | Consultant Anaesthetist. |
| Dallas, N. L., M.A., M.B., B.CHIR., F.R.C.S.E. | Consultant Ophthalmic Surgeon. |
| Roylance, J., M.B., CH.B., D.M.R.D. | Consultant Radiologist. |
| Burman, D., B.SC., M.D., B.S., D.C.H., M.R.C.P. | Consultant Paediatrician. |
| Faulkner, D., M.B., CH.B., F.F.A.R.C.S., D.A. | Senior Registrar in Anaesthetics. |
| Clement, J. A., M.B., B.S., D.A. | Senior Registrar in Anaesthetics. |
| Lall, M., M.B., B.S., F.R.C.S. | Senior Registrar in E.N.T. |
| Morgan, N. V., M.A., B.M., B.CH. | Senior Registrar in E.N.T. |
| Henderson, A. H., B.A., M.B., B.CHIR., M.R.C.P. | Senior Registrar in Medicine. |

| | |
|---|---|
| Vickery, C. M., M.B., B.S., F.R.C.S. | Senior Registrar in Surgery. |
| Bicknell, M. R., M.B., B.S. | Senior Registrar in E.N.T. |
| Roberts, J. B. M., M.B., CH.B., F.R.C.S. | Senior Registrar in Surgery. |
| Blecher, T. E., M.B., B.CH., M.R.C.P. (Ed.). | Senior Registrar in Pathology. |
| Waters, T. C., M.B., CH.B. | Senior Registrar in Psychiatry. |
| Roberts, J. H., M.B., CH.B., D.M.R.D. | Senior Registrar in Radiology. |
| Davies, J., M.B., CH.B., F.F.A.R.C.S., D.A. | Senior Registrar in Anaesthetics. |
| Walker, W. H. C., M.B., B.S. | Senior Registrar in Chemical Pathology. |
| Harley, A., M.B., B.CHIR. | Registrar in Medicine. |
| Thirkettle, J. L., M.B., B.S., M.R.C.P. | Registrar in Medicine. |
| Keir, M. I. S., M.B., B.CH. | Registrar in Dermatology. |
| Powell, J. N., M.B., B.CHIR., F.F.A.R.C.S., D.A. | Registrar in Anaesthetics. |
| Taranto, B., M.B., B.S. | Registrar in Ophthalmology. |
| Edwards, N., M.B., B.CHIR. | Registrar in E.N.T. |
| Keane, P. M., M.B., CH.B. | Registrar in Pathology. |
| Roome, A. P. C. H., M.B., CH.B. | Registrar in Pathology. |
| Beddard, J. B., M.B., CH.B. | Registrar in Anaesthetics. |
| Clough, J. R., M.B., B.S., F.R.C.S. | Registrar in Orthopaedics. |
| Kiff, J. H., B.SC., M.B., B.S. | Registrar in Anaesthetics. |
| Murphy, N. B., M.B., B.CH., B.A.O., N.U.I. | Registrar in Radiology. |
| Evison, G., M.B., CH.B. | Registrar in Radiology. |
| Forbes, W. R., M.B., B.CH., B.A.O., D.C.H. | Registrar in Paediatrics. |
| Kamath, R. V. | Registrar in Cardiology. |
| Mercer, D., M.B., CH.B. | Registrar in Radiotherapy. |
| Diamond, A. W., M.B., B.S. | Registrar in Anaesthetics. |
| Lambert, T. F., M.B., CH.B. | Registrar in Anaesthetics. |
| Buckler, K. G., M.B., CH.B., F.R.C.S. | Registrar in General Surgery. |
| Tandon, R. K., M.B., B.S. | Registrar in Thoracic and Cardiac Surgery. |
| Pantazopoulos, T. | Registrar in Orthopaedics. |
| Louisy, C. L., M.B., CH.B. | Registrar Trainee in Radiology. |
| Mitchell, A. V., M.B., B.CH., B.A.O. | Registrar Trainee in Radiology. |
| Gibson, M. J., B.SC., M.B., CH.B. | Registrar Trainee in Radiology. |

*Printed by*

J. W. Arrowsmith Ltd., Bristol

